# Post-COVID-19 Neurological Sequelae of Polyneuropathy and Encephalitis: A Comprehensive Case Report

**DOI:** 10.7759/cureus.56645

**Published:** 2024-03-21

**Authors:** Sebastian L Manuel, Jenna Sapone, Frank Lin, Mathai Chalunkal

**Affiliations:** 1 Internal Medicine, St. Luke's University Health Network, Easton, USA

**Keywords:** behavioral changes, upper limb paralysis, lower limb paralysis, seronegative autoimmune encephalitis, polyneuropathy, covid-19

## Abstract

Severe acute respiratory syndrome coronavirus 2 (SARS-CoV-2) infection, commonly known as COVID-19, has been associated with various neurological complications. However, the mechanisms underlying these neurological manifestations remain incompletely understood. We present a case of a 63-year-old male who was admitted to the intensive care unit with severe COVID-19 pneumonia. Following recovery from respiratory symptoms, he was found to have weakness in the limbs. Months later, he also developed altered mental status, hallucinations, and behavioral changes. Neurological examination revealed signs consistent with polyneuropathy and autoimmune encephalitis. Further investigations, including nerve conduction studies, cerebrospinal fluid analysis, and response to steroids, supported the diagnosis of COVID-19-related polyneuropathy and autoimmune encephalitis. This is a rare presentation of COVID-19 and has only been described in a few case reports. Further research is warranted to elucidate the pathophysiological mechanisms underlying neurological sequelae of COVID-19 and to develop targeted therapeutic strategies.

## Introduction

The novel coronavirus SARS-CoV-2 is commonly known to affect the respiratory system, but other organ systems, including the nervous system, can become dysfunctional as a result of severe illness. Central nervous system (CNS) manifestations like stroke, seizures, venous sinus thrombosis, meningitis, encephalitis, myelitis, and CNS vasculitis, and peripheral nervous system (PNS) manifestations like Guillain-Barre syndrome (GBS) and myopathy have been reported with COVID-19 infection [1,2]. Even with these known complications associated with severe COVID-19 infection, it is often difficult to distinguish whether the neurological features are manifestations of critical illness or specific to the virus itself. Here, we present a unique case of CNS polyneuropathy and encephalitis, after a severe case of COVID-19 infection.

## Case presentation

A 63-year-old male with an extensive past medical history of coronary heart disease, type 2 diabetes, hypertension, hyperlipidemia, peripheral vascular disease, chronic kidney disease stage 3, and heart failure with reduced ejection fraction was admitted for severe COVID-19. He required intubation for acute respiratory failure and was admitted to the intensive care unit (ICU). He was placed on a severe COVID-19 pathway, where he completed a 10-day course of intravenous (IV) dexamethasone, a five-day course of remdesivir, and one dose of convalescent plasma. His course was complicated with encephalopathy, which was thought to be secondary to ICU delirium, hypoxia, or uremia. Computed tomography (CT) of the head at this time was negative for any acute intracranial abnormalities. His stay was then further complicated with an acute GI bleed from a gastric ulcer, where hemostasis was achieved after esophagogastroduodenoscopy (EGD) interventions. The patient continued to be severely encephalopathic, which inhibited extubation despite multiple spontaneous breathing trials. Due to his prolonged intubation and persistent encephalopathy, the patient ultimately required tracheostomy and percutaneous endoscopic gastrostomy (PEG) placement.

After 19 days of hospitalization, his mentation slightly improved and he began following minimal commands but remained globally weak. His right upper extremity and hand strength were significantly decreased. He was unable to lift his right arm and forearm against gravity. Repeat CT of the head remained negative for any intracranial abnormalities. Global weakness of all extremities was noted. A diagnosis of critical illness polyneuropathy and myopathy was made clinically. It was determined that no additional neurologic workup was warranted, and neurology recommended outpatient follow-up. The patient’s overall physical strength remained severely compromised and he was dependent on his caregivers for all his activities of daily living in the rehabilitation facility. He was wheelchair-bound. With months of physical and occupational therapy, the patient had minimal improvements. Tables 1, 2 demonstrate the neurological physical exam during outpatient follow-up, five months after discharge. There was asymmetrical weakness noted with the right distal extremities being more affected than the left. Distal lower extremities were equally weak. There were no sensory deficits.

**Table 1:** The patient's neurological exam findings CN: cranial nerve.

Neurological exam			
Appearance: no acute distress			
				Ophthalmoscopic: disc flat, normal fundus			
Mental status exam	Orientation: awake, alert, and oriented x3						
	Memory: registration 3/3, recall 3/3		
	Attention: normal; knowledge: good		
	Language: no aphasia; speech: no dysarthria		
Cranial nerves exam	CN 2: no visual defect on confrontation, pupils round, equal, reactive to light		
	CN 3, 4, and 6: extraocular movements intact, no nystagmus		
	CN 5: facial sensation intact		
	CN 7: no facial asymmetry		
	CN 8: intact hearing		
	CN 9 and 10: palate symmetric, normal gag		
	CN 11: good shoulder shrug		
	CN 12: tongue midline		

**Table 2:** Patient's motor and sensory physical examination BR: brachioradialis tendon reflex; B: bicep tendon reflex; T: triceps tendon reflex; P: patellar tendon reflex; A: Achilles reflex.

Muscle strength testing					
Upper extremity			Lower extremity		
	Right	Left		Right	Left
Deltoid	4/5	4/5	Iliopsoas	4/5	4/5
Biceps	4/5	4/5	Quads	4/5	4/5
Triceps	4/5	4/5	Hamstrings	4/5	4/5
Wrist extension	2/5	4/5	Ankle dorsi flexion	3/5	2/5
Wrist flexion	2/5	4/5	Ankle plantar flexion	4/5	3/5
Interossei	1/5	4/5	Ankle eversion	3/5	3/5
Abductor pollicis brevis	1/5	4/5	Ankle inversion	2/5	2/5
Deep tendon reflexes					
	BR	B	T	P	A
Right	2+	2+	2+	2+	0
Left	2+	2+	2+	2+	0
Sensory: no sensory deficits. Symmetric pinprick, light touch, vibration, and proprioception					
Gait: stable					
Coordination: no ataxia with finger-to-nose testing and heel-to-shin					

Further diagnostic studies were completed, which included magnetic resonance imaging (MRI) of the brain and cervical spine, and electromyography (EMG). MRI of the brain ruled out acute stroke or intracranial abnormalities. MRI of the cervical spine demonstrated degenerative disease with multi-spinal canal narrowing, unlikely related to the disease course. EMG impression revealed diffuse severe axonal motor polyneuropathy in the upper and lower extremities and evidence to support chronic and active denervation in the upper and lower extremity distal motor muscles. The neurologist attributed these findings to post-COVID-19 neurological sequelae as well as critical illness polyneuropathy. The patient returned to his rehabilitation facility and continued physical and occupational therapy for over a year after discharge. He was never able to regain his strength and remained dependent on his family for his activities of daily living.

Unfortunately, the patient continued to have multiple emergency department visits and admissions for intermittent episodes of encephalopathy and neck pain. A lumbar puncture revealed an elevated protein of 108 mg/dL. Cerebrospinal fluid (CSF) gram stains and cultures were negative. Anti-Hu antibodies, anti-Ri antibodies, and Venereal Disease Research Laboratory (VDRL) tests were negative. Autoimmune workup, including C3, C4, and anti-neutrophil cytoplasmic antibody (ANCA) levels, was also negative. Serum immunoglobulin (Ig) kappa free light was high at 137.0 mg/L and Ig lambda free light chain was also high at 91.5 mg/L. An electroencephalogram (EEG) revealed no seizure activity. Theta frequency and background slowing suggested mild nonspecific diffuse cerebral dysfunction. Differentials at this time included autoimmune encephalitis versus post-COVID encephalitis. He was then treated with five days of intravenous immunoglobulin (IVIG) and oral prednisone. Mentation and hallucinations significantly improved after treatment. Unfortunately, this patient suffered additional hospitalizations and progressively worsened due to the high severity of other comorbidities, which led to his eventual passing.

## Discussion

This case report shows an association between severe COVID-19 infection and the development of neurological sequelae. Our particular patient developed post-COVID-19 polyneuropathy and encephalopathy. The polyneuropathy was evidenced by EMG findings that showed diffuse severe axonal motor polyneuropathy in the upper and lower extremities (more pronounced in the distal extremities compared to the proximal). The encephalopathy was evidenced clinically by behavioral changes, hallucinations, and elevated proteins in lumbar puncture.

A diagnosis of COVID-19 neurological manifestations requires the exclusion of alternate etiologies, including sepsis, metabolic, autoimmune, or critical illness neuropathy. Sepsis alone can result in encephalopathy or polyneuropathy in 70% of patients [2]. Critical illness neuropathy is a sensorimotor polyneuropathy that is often a complication of sepsis and multiorgan failure, occurring in 70% of such patients [3]. Flaccid weakness of the extremities and loss of tendon reflexes are associated findings of critical illness polyneuropathy and myopathy. The severe weakness attributed to both critical illness neuropathy and myopathy can require mechanical ventilation. Here, we present a unique case of a patient treated in the intensive care unit who was found to have a severe acute COVID-19 infection complicated by post-COVID and critical illness polyneuropathy.

Previous studies have associated COVID-19 with PNS involvement. A meta-analysis by Hanganu et al. found that PNS involvement was the most commonly associated with GBS. In this study, 429 (42.7%) patients had non-severe forms of COVID-19, and 133 (13.2%) had severe forms of the disease [4]. GBS caused by COVID-19 was often described as progressive, ascending limb weakness that evolves throughout one to four days [5]. The onset of weakness usually starts five to 16 days after infection [6]. The patient did not present with ascending limb weakness, which makes GBS less likely. One case report described the involvement of the bilateral peroneal nerve, which presented with zero ankle reflexes and foot drop bilaterally [7]. Critical illness polyneuropathy and myopathy were also considered part of the differentials for the patient; however, the muscle weakness was not symmetrical, and there were no sensory deficits noted [8]. Our patient's right arm was significantly weaker than the left.

Interestingly, months after the COVID-19 infection, the patient developed behavioral changes, waxing and waning mental status, hallucinations, and headaches. Repeat MRI of the brain and autoimmune workup were negative. Research has suggested that the pathophysiology remains a gray area; however, it has been hypothesized it could either be due to direct damage of neurological tissues or indirectly through immune-mediated mechanisms [9]. One case report described a patient who had multiple seizure episodes after recovering from COVID-19 infection [9]. In that case, imaging showed a left frontal gyrus lesion. CSF showed lymphocytic pleocytosis, elevated protein, and elevated IgG, which favored an inflammatory process. Similar to our case, the patient also clinically improved after receiving IVIG.

Most cases of autoimmune encephalitis from COVID-19 were found to be positive for specific antibodies, such as anti-N-methyl-D-aspartate receptor (NMDAR) antibodies (most common), anti-myelin oligodendrocyte glycoprotein (MOG) antibody encephalitis, anti-amphiphysin antibodies, and contactin-associated protein (Caspr2) antibodies [10]. Some cases had no identifiable antibodies [10]. Our case also had no identifiable antibodies.

The presentation seems to fit the criteria created by Graus et al. to diagnose autoimmune encephalitis: our patient had a rapid progression of symptoms that included altered mental status and psychiatric symptoms, exclusion of other autoimmune encephalitis syndromes, CSF pleocytosis in the lumbar tap, and reasonable exclusion of other causes [11]. The case was further supported by improvement with IVIG and steroids.

## Conclusions

This case report highlights the emergence of neurological complications, specifically polyneuropathy and encephalitis, in patients recovering from severe COVID-19 infection. Clinicians should remain vigilant for such neurological manifestations in individuals with a history of COVID-19, especially those experiencing lingering symptoms or new-onset neurological deficits. Timely recognition and management are crucial to prevent long-term sequelae and optimize patient outcomes. Further research is warranted to elucidate the underlying mechanisms and risk factors contributing to post-COVID-19 neurological complications.
